# Supplementary material for: Splice-Junction-Based Mapping of Alternative Isoforms in the Human Proteome
Source: Cell Rep. Author manuscript; Available in PMC 2020 Jan 15. (PMC6961840; doi:10.1016/j.celrep.2019.11.026)
Supplement: 2 [file NIHMS1546469-supplement-2.zip › DF1/PXD010154/Colon/Colon_17_SHPRH_HSEASVLTVADLADLFTKETEELYPFTF.pdf]

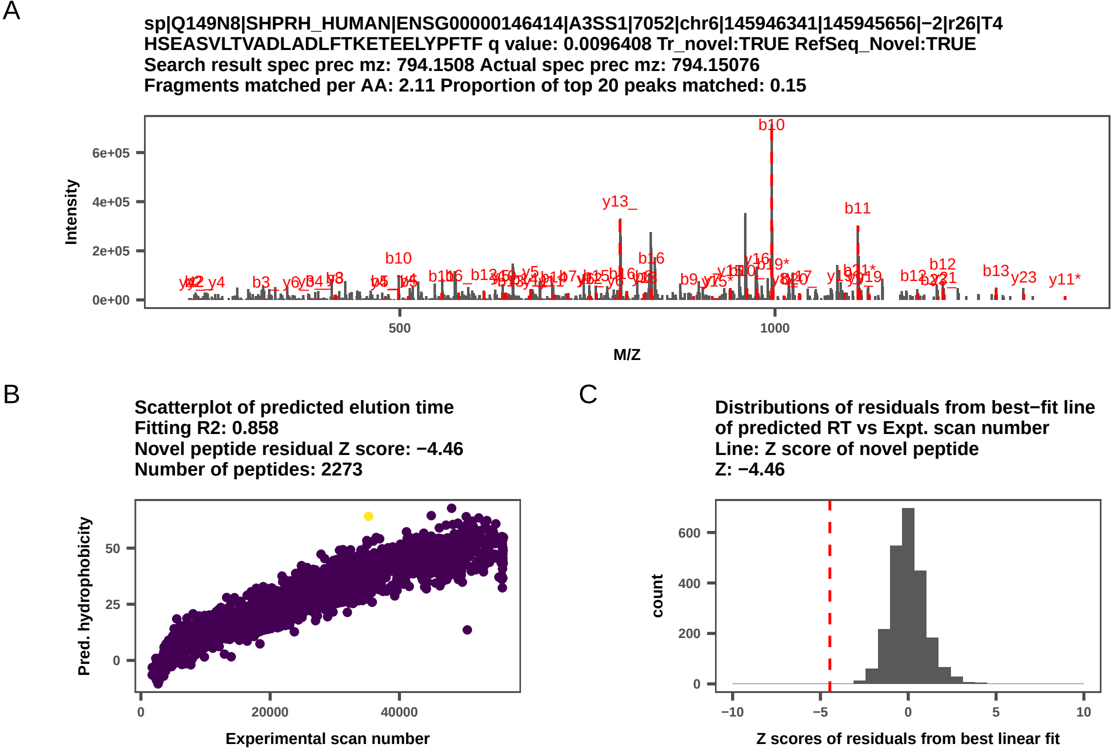
A
sp|Q149N8|SHPRH_HUMAN|ENSG00000146414|A3SS1|7052|chr6|145946341|145945656|−2|r26|T4
HSEASVLTVADLADLFTKETEELYPFTF q value: 0.0096408 Tr_novel:TRUE RefSeq_Novel:TRUE
Search result spec prec mz: 794.1508 Actual spec prec mz: 794.15076
Fragments matched per AA: 2.11 Proportion of top 20 peaks matched: 0.15
Intensity
6e+05
4e+05
2e+05
0e+00
b10
y13_
b11
b16
b12
b13
y23
y11*
b3
b10
500
1000
M/Z
B
Scatterplot of predicted elution time
Fitting R2: 0.858
Novel peptide residual Z score: −4.46
Number of peptides: 2273
Pred. hydrophobicity
50
25
0
0
20000
40000
Experimental scan number
C
Distributions of residuals from best−fit line
of predicted RT vs Expt. scan number
Line: Z score of novel peptide
Z: −4.46
count
600
400
200
0
−10
−5
0
5
10
Z scores of residuals from best linear fit
